# Cybergenetic control of microbial community composition

**DOI:** 10.3389/fbioe.2022.957140

**Published:** 2022-10-06

**Authors:** Ting An Lee, Harrison Steel

**Affiliations:** Department of Engineering Science, University of Oxford, Oxford, United Kingdom

**Keywords:** microbial community, composition, microbiome, quantitative, synthetic biology, cybergenetics, *in silico* control

## Abstract

The use of bacterial communities in bioproduction instead of monocultures has potential advantages including increased productivity through division of labour, ability to utilise cheaper substrates, and robustness against perturbations. A key challenge in the application of engineered bacterial communities is the ability to reliably control the composition of the community in terms of its constituent species. This is crucial to prevent faster growing species from outcompeting others with a lower relative fitness, and to ensure that all species are present at an optimal ratio during different steps in a biotechnological process. In contrast to purely biological approaches such as synthetic quorum sensing circuits or paired auxotrophies, cybergenetic control techniques - those in which computers interface with living cells-are emerging as an alternative approach with many advantages. The community composition is measured through methods such as fluorescence intensity or flow cytometry, with measured data fed real-time into a computer. A control action is computed using a variety of possible control algorithms and then applied to the system, with actuation taking the form of chemical (e.g., inducers, nutrients) or physical (e.g., optogenetic, mechanical) inputs. Subsequent changes in composition are then measured and the cycle repeated, maintaining or driving the system to a desired state. This review discusses recent and future developments in methods for implementing cybergenetic control systems, contrasts their capabilities with those of traditional biological methods of population control, and discusses future directions and outstanding challenges for the field.

## Introduction

A microbial community consists of two or more co-occurring species within a defined environment. As community function is the cumulative product of all individuals within the community, the ability to control the composition and relative abundance of species in synthetic and natural communities is highly desirable. Dysbiosis (defined here as a reduction in diversity and loss of species with “beneficial” effects) of natural communities such as the human gut microbiome or plant rhizosphere can have profound effects on their host as a result of altered community function, and has been implicated in conditions such as cancer (Xavier et al., 2020) or decreased plant growth (Yin et al., 2021). Population control is also crucial for applications of synthetic consortia in biotechnology, which have received increased interest for their potential advantages over monocultures. These include (for example) the ability to utilise more complex substrates (Xia, Eiteman and Altman, 2012), division of labour for decreased metabolic burden and cross-talk between parts (Tsoi et al., 2018), and increased yield and robustness to environmental fluctuations (Stenuit and Agathos, 2015). However, without a method for maintaining a community’s composition, even minor fitness differences between species can cause some to outcompete and overwhelm other species, destabilising the community and leading to decreased efficiency or population collapse.

**FIGURE 1:**
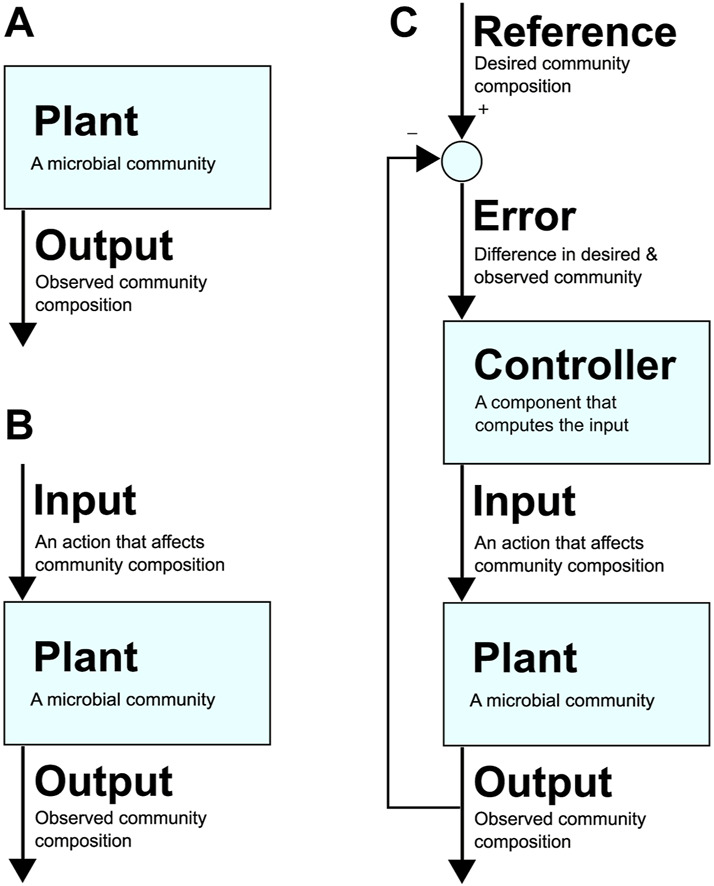
**(A)** An uncontrolled system. The community (which can be a synthetic or natural community in any environment) is the “plant”. Its composition at a given time is the plant’s “output”. **(B)**: A system with open-loop control. A control action or “input” to the plant causes a change in the output. **(C)**: A system with closed-loop control with a feedback loop. The desired composition at a given time is the “reference”, and the difference between the reference and output is the “error”, which is used by the “controller” to decide on “input” that affects community composition in a way that drives the error towards zero (i.e., towards desired community composition).

To realise the immense potential benefits of synthetic consortia, researchers are beginning to build upon robust control methods that are foundational to the many fields of applied engineering and technology that leverage tools from control theory. Figure 1 presents an example architecture of a feedback control loop applied to a microbial community. To use the lexicon of the field, here the microbial community is referred to as the “plant” (i.e., the part of the system that needs to be controlled). Its composition at a particular instance is the system’s “output”, while the desired community composition is the “reference”. An uncontrolled system is represented by only a plant and its output (Figure 1A), while an open-loop system also includes an “input”, which is an action taken to alter the community composition (e.g., adding an antibiotic that targets one species) and has no feedback in response to the output (Figure 1B). In contrast, a closed-loop system defines an “error” as the difference between the output and reference, i.e., the difference between desired and observed community composition (Figure 1C). Closing the loop involves a “controller” that computes an input for the plant that attempts to reduce the error over time, eventually bringing it to zero, i.e., where the community’s current composition is identical to the desired composition.

Recent work has delivered several examples of biologically-implemented compositional control of co-cultures (two species communities), as well as larger communities where there is no external input and all aspects of the system such as the controller, method of measuring output [e.g., through quorum sensing (Scott et al., 2017)], and method of altering composition [e.g., expression of bacteriocins (Kong et al., 2018)] are implemented biologically. This is covered in more depth in several reviews: McCarty and Ledesma-Amaro (2019), Grandel, Reyes Gamas and Bennett (2021), Perrino et al. (2021), and Ronda and Wang (2022). Applying a control theory perspective to biological approaches to controlling microbial communities highlights possible potential areas of improvement, which are organised here into limitations in performance or capabilities, and difficulties in implementation.

First, one of the capabilities that many biologically controlled communities lack is the ability to implement a dynamic reference i.e., where the desired composition is not static over time. This is highly desirable in situations where the “optimum” composition is not constant: for example, in a community where a pathway of interest is distributed between species (Dinh, Chen and Prather, 2020; Salma et al., 2021) (e.g., to reduce metabolic burden), the bottleneck may shift over time, for which an optimal controller could compensate. Additionally, even in systems where the biological control circuit can create oscillating or dynamic community compositions (Chen et al., 2015), because it is encoded genetically into cells, changing the reference is difficult to do (typically requiring tuning expression of components in the biological control circuit or restarting the culture with adjusted initial inoculation ratios), necessitating many laborious iterations to probe different community compositions (Jones et al., 2017). This makes it infeasible to optimise and rapidly explore the design space by searching for compositions that are best at a desired function (e.g., highest yield), analogous to how combinatorial DNA assembly can be used to tune promoter and ribosome binding site strength for optimal production (Storch et al., 2017). Finally, biologically controlled systems are complex and influenced by factors such as stochasticity or uncertain operating conditions. For example, approaches that depend on the concentration of quorum sensing signals (You et al., 2004; Balagaddé et al., 2008; Kong et al., 2018; Stephens et al., 2019; Miano, Liao and Hasty, 2020) are susceptible to variations that may arise from a change in growth phase and may not be consistent in different environments (e.g., batch vs. controlled exponential growth or small vs. large scale cultures), which can degrade or break the control approach. With the control approach genetically encoded, there is also little opportunity for intervention and human input to adjust the approach to counteract stochasticity or changing conditions.

Second, building and implementing complex biological controllers remains significantly challenging. Initial system design requires the selection of appropriate biological parts (e.g., inducible promoters) with known characteristics (e.g., dynamic range, basal output) that can produce the desired control scheme. This problem is exacerbated when trying to work with larger communities or those containing non-model organisms, which may lack toolboxes of well-characterised parts. Crucially, parts within each species (e.g., promoters) and parts for interspecies communication (e.g., quorum sensing molecules) need to be orthogonal, and even then are susceptible to cross talk through the use of shared cellular resources (Zhang et al., 2021). While new orthogonal quorum sensing systems are emerging (Scott and Hasty, 2016; Kylilis et al., 2018; Jiang et al., 2020), it remains a factor preventing the design and control of arbitrarily large communities. These parts may also not be easily transferred to different species (Adams, 2016), preventing circuits that worked in one community from being translated into another. Past the design stage, biological control circuits face further challenges when engineered into cells: while it is possible to encode increasingly complex logic circuits (Nielsen et al., 2016), they contain more possible points of failure (Fontanarrosa et al., 2020) and come at the cost of increased metabolic burden (Borkowski et al., 2016), resulting in 1) strong selective pressure for mutations that alleviate the burden, but consequently break the control scheme, 2) diversion of cellular resources from the product/function the cell is responsible for, possibly negating the benefits of a community, or 3) altered circuit dynamics because individual parts do not function as intended. An early example of biologically controlled communities, a two species predator-prey *E. coli* co-culture where LuxR and LasI quorum sensing molecules were linked to expression of the ccdB toxin, could not be implemented at macroscale as circuit function was lost before it could be observed (Balagaddé et al., 2008). The desired behaviour was observed in microchemostats with a 9 nl volume, which required fewer cell divisions and thus was less vulnerable to the burden imposed by the circuit. While later examples of biologically controlled communities have been implemented at a macroscale, burden is exacerbated when the control circuit is expanded-whether to control communities with more species or perform more complex control functions.

Similar issues to the above can arise for those attempting to control any biological system. The next section will therefore highlight systems where biology is interfaced with computer control and how it overcomes some of these issues; the rest of the review will then explore its application to microbial communities.

## Cybergenetic control

**FIGURE 2:**
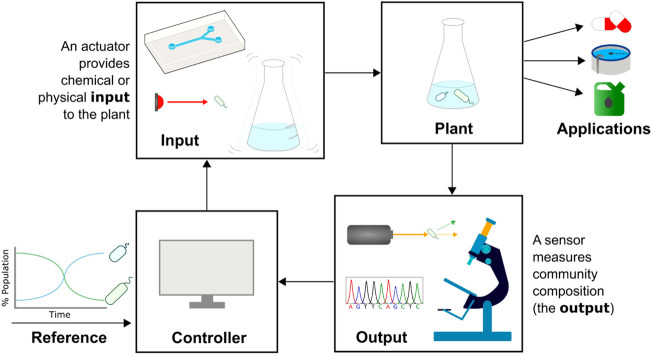
Cybergenetic control of community composition. The composition of a microbial community (plant, top right) needs to be controlled for optimal function, e.g., bioproduction or bioremediation. A sensor such as a flow cytometer, sequencer, or microscope is used to measure community composition in real-time (output). The measured and desired population data is fed into a computer acting as the controller, which decides on a control action. An actuator provides a chemical (e.g., injecting media with chemical inducers into a microfluidic device) or physical (e.g., shining light or shaking the culture vessel) input to the plant that leads to a change in community composition, and the cycle is repeated.

Cybergenetic control describes the interfacing of biological systems with computers to create hybrid systems that bring together the best features of biological and computational engineering (Figure 2). A system’s output (e.g., expression of a fluorescent protein) is measured and processed by a computer, which uses the output data and a control strategy to decide on an input. The input is then applied to the community *via* an automated actuation device (e.g., a syringe pumping a chemical inducer), closing the feedback loop. In the application of this field to the control of gene expression, computers control the expression of genes such as fluorescent reporters in single cells or monocultures, and has been used to explore gene networks (Menolascina et al., 2014), understand regulatory dynamics (Rullan et al., 2018), and tune growth rates (Milias-Argeitis et al., 2016). Such examples and the methods used are covered in reviews by Carrasco-López et al. (2020) and Lugagne and Dunlop (2019). In this paper we will build on these ideas, describing recent progress in the field of monitoring and actuating microbial communities and the application of cybergenetic control to cells within a community, rather than genes within a cell.

By implementing control actions electronically, cybergenetic control can address many issues facing biological control. In terms of performance, it enables dynamic and temporally complex reference compositions rather than a single steady state that is genetically hard-coded. The system’s decision-making circuitry is unaffected by biological fluctuations such as growth phase, substrate/product concentrations, or culture size, and can rapidly adjust its control approach to respond to fluctuations, allowing for robust control in different contexts. Additionally, while it cannot completely negate the effects of evolution (since cells can mutate to ignore inputs or stop providing measurable outputs), the control approach can be adjusted in real-time to deal with minor mutations (e.g., if a species evolves to become less responsive to an input, the duration or intensity of an input can increase). In terms of implementation, computing the control approach *in silico* removes the need for control circuits that scale in size with community complexity, partially mitigating issues such as retroactivity or the need for orthogonal parts. The decreased metabolic burden also reduces the selective pressure to evolve away from the control circuit.

Even if biological capabilities advanced rapidly (e.g., increased availability of orthogonal parts, methods of alleviating burden), as highlighted by Kumar, Rullan and Khammash (2021), a common control engineering strategy in fields such as aerospace is “Hardware-In-the-Loop” (HIL) testing, where a controller is connected to a test system that simulates reality. It allows for design and optimisation of controllers in systems where iterative testing is otherwise too time consuming or expensive, accelerating the design cycle. Cybergenetic control represents the application of this strategy to biology, interfacing cells with computers to better understand the system, build and test models and hypotheses, and rapidly iterate and probe the design space to design and optimise a control approach before finally implementing it biologically. For example, similar to how knockouts can be used to probe genes responsible for a phenotype, cybergenetic control could facilitate the testing of biological hypotheses such as links between community structure and function (Bell, 2019). As such, even if biologically-implemented control is the ultimate goal, cybergenetic control may be a suitable and beneficial intermediate step toward technological maturation.

To implement cybergenetic control (Figure 2), a system requires an input method (actuating one or several species in the community to affect their relative abundance), an output (measuring the composition), and a control algorithm that describes what input should be provided to achieve a desired output, given the current state of the system. Subsequent sections highlight existing methods for each of these aspects, discussing factors to consider and which are most suitable in different circumstances.

## Control input: Actuating species in a community

**FIGURE 3:**
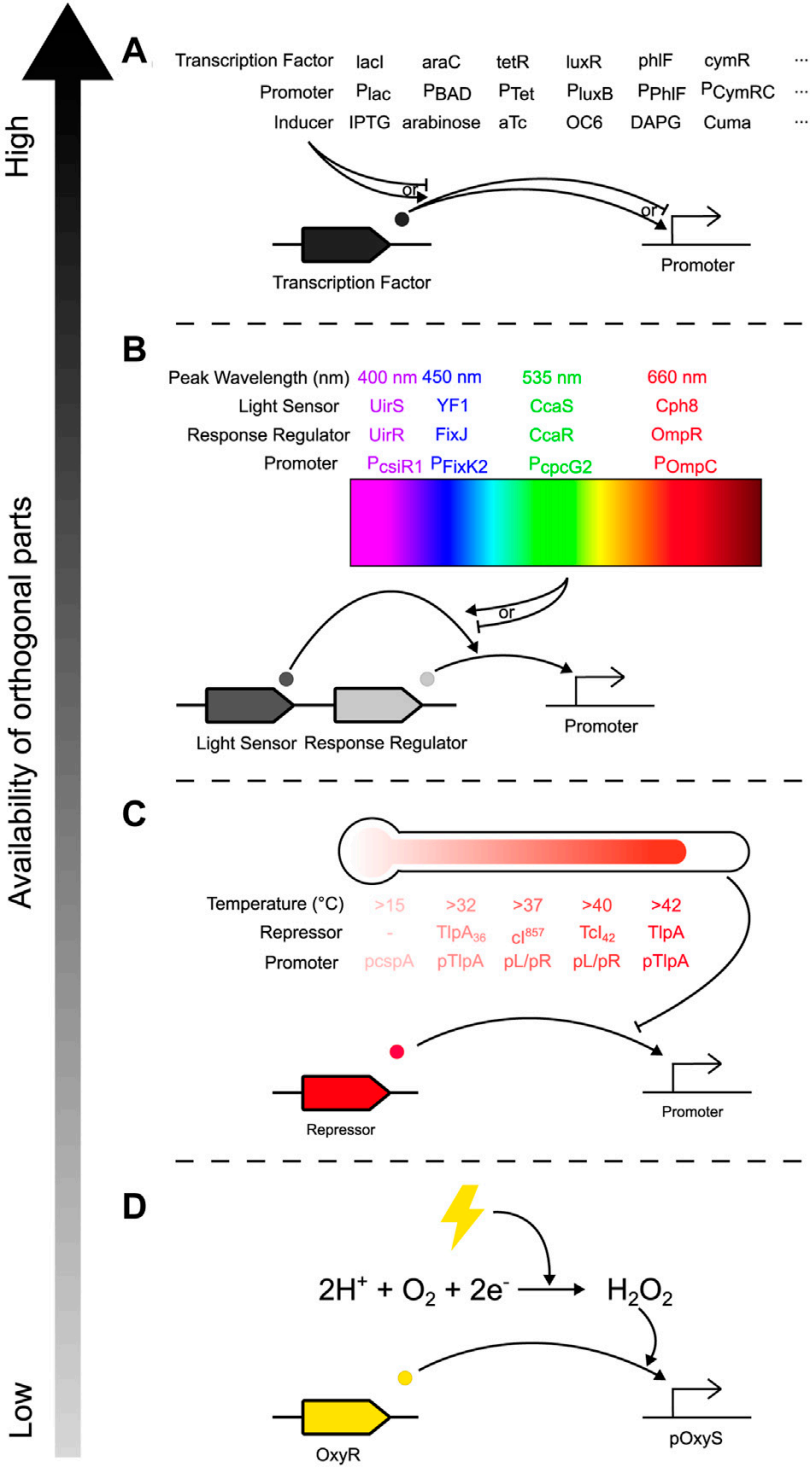
Toolbox of orthogonal genetic parts for inducing gene expression in bacterial communities, arranged by availability of orthogonal parts from highest to lowest. Common examples from each input method are depicted, but this is not an exhaustive list, and other mechanisms and implementations are possible for each method. **(A)** Chemically induced promoters, where expression of a gene of interest (GOI) is activated/inhibited by a transcription factor, which is itself activated/inhibited by exogenous addition of a chemical inducer. **(B)** Optogenetic regulators (here illustrated by two-component systems), where a light-sensing protein is reversibly changed after exposure to particular wavelengths of light and activates a response-regulating transcription factor that activates expression of a GOI. **(C)** Temperature sensitive promoters, which are typically repressed by a transcription factor whose activity is diminished past a certain temperature, activating expression of a GOI. **(D)** Peroxide inducible promoters which respond to electrical inputs. Application of electricity to the environment reduces dissolved oxygen to hydrogen peroxide, which activates the OxyR transcriptional activator, thus enabling expression of a GOI.

Controlling community composition requires control inputs that can affect the relative abundance of constituent species. This can be achieved through several methods, one of which is engineering species to have a change in abundance in response to an external signal. This requires regulatory components that can transduce the signal into the cell and an actuating component that causes the change in abundance. There is an ever-increasing toolbox of novel genetic parts that can transduce signals into cells: a wide variety of chemically inducible promoters have been developed (Examples in Figure 3A), 12 of which were demonstrated to be orthogonal when integrated into a single strain of *E. coli* (Meyer et al., 2019), while new promoters can be mined and developed for other species in the community (Weinstock et al., 2016). Exogenous addition of chemical inducers can be automated: at microscale, computer-controlled syringes can pump chemicals into a microfluidic chamber (Figure 2, Input) (Menolascina et al., 2014; Burmeister and Grünberger, 2020; Burmeister et al., 2021); while at macroscale, cheap bioreactor systems such as Chi.Bio (Steel et al., 2020), the turbidostat designed by Guarino et al. (2019), or the turbidostat of the eVOLVER platform (Wong et al., 2018; Gutiérrez, Kumar and Khammash, 2022) removes the need for manual input without expensive or custom-built equipment.

Alternatively, light is another common method of inducing gene expression (LED in Figures 2, 3B) (Levskaya et al., 2005; Hirose et al., 2008; Möglich, Ayers and Moffat, 2009; Toettcher et al., 2011; Melendez et al., 2014; Baumschlager, Aoki and Khammash, 2017; Chait et al., 2017; Rullan et al., 2018; Li et al., 2020; Baumschlager and Khammash, 2021; Lalwani et al., 2021; Lindner and Diepold, 2022; Sheets and Dunlop, 2022). Optogenetic approaches in bacteria [reviewed by Baumschlager and Khammash (2021)] such as one- and two-component systems (Figure 3B) or photocaged and photosensitive inducers have several benefits: application of different wavelengths of light is instantaneous, unlike chemical inducers which have delays during pumping and mixing/diffusing through the culture; it is quickly reversible while chemicals are persistent and need to be consumed or diluted out, allowing for more temporally complex induction profiles; it allows for spatially patterned induction; and while high-intensity light can have phototoxic effects (Baumschlager and Khammash, 2021), light has less potential for off-target effects (e.g., compared to many chemical inducers). Light can also be used in conjunction with chemical approaches such as with photocaged IPTG (Isopropyl β-D-1-thiogalactopyranoside), which can be transported into cells with no effect and then uncaged with UV-A light for faster and more homogenous induction (Burmeister et al., 2021), or inactivation of aTc (anhydrotetracycline), pausing transcription without the need to dilute all the aTc from the growth media (Baumschlager, Rullan and Khammash, 2020). However, while light can be implemented at small to medium scales, light penetration may be a problem for large, dense cultures, and is difficult to implement in certain natural environments such as the gut. Additionally, while many optogenetic systems have been developed and described on databases like Optobase (Kolar et al., 2018), light input is limited by the number of orthogonal light-sensitive parts which respond to non-overlapping wavelengths, constraining the complexity of a community that can be controlled exclusively with light. Other response-inducing inputs include: promoters sensitive to temperature (Figure 3C) allowing actuation of communities within human tissue through localised heating using focused ultrasound (Vasina, Peterson and Baneyx, 1998; Valdez-Cruz et al., 2010; Hoynes-O’Connor et al., 2015; Piraner et al., 2017; Piraner, Wu and Shapiro, 2019; Zheng et al., 2019); osmolarity (Uhlendorf et al., 2012), and electricity through (for example) peroxide-inducible promoters that sense electrochemical reduction of oxygen to hydrogen peroxide (Figure 3D) (Terrell et al., 2021). However, these methods have distinct disadvantages against chemical and light induction: while there are orthogonal options (e.g., promoters activated at a range of temperatures/osmolarities), these options are not as easily multiplexable (e.g., several chemical inducers can be added concurrently, but temperature can only be a single value at a given time). These methods may also have off-target effects; compared to a monoculture where temperature induces a response in a homogenous population, in a community temperature change may differentially affect constituent species (e.g., select for cold-tolerant bacteria).

Coupled to these regulatory parts are components that actuate cell behaviour to affect relative abundance. For example, a species’ relative abundance can be decreased by initiating cell death [e.g., by expressing a lysis gene such as ccdB (Balagaddé et al., 2008; Scott et al., 2017)] or growth arrest (Edwards et al., 2013; Aditya et al., 2021). In contrast, expression of toxins or anti-microbial peptides such as bacteriocins targeting other species (Kong et al., 2018; Liu et al., 2019; Fedorec et al., 2021; Lalwani et al., 2021) or expression of genes that enable growth (e.g., antibiotic resistance cassettes or anti-toxins) (Liu et al., 2019; Lalwani et al., 2021; Gutiérrez, Kumar and Khammash, 2022; Sheets and Dunlop, 2022) will increase the species’ relative abundance instead. For example, Lalwani et al. (2021) demonstrated open loop control of an *E. coli*-*S. cerevisiae* co-culture through optogenetic regulation of the MazEF toxin-antitoxin system. Fine-tune adjustments for a more graded response could be achieved by altering a species’ fitness and growth rate e.g., adjusting metabolic burden or placing enzymes such as RNA polymerase (Izard et al., 2015), amino acid synthases (Milias-Argeitis et al., 2016) or those responsible for central glucose metabolism (Stephens et al., 2019; Dinh, Chen and Prather, 2020) under inducible control. Inputs can also be used to adjust the strength of interspecies interactions (e.g., tuning the expression of quorum sensing molecules that species use to communicate with each other (Balagaddé et al., 2008; Dinh, Chen and Prather, 2020; Miano, Liao and Hasty, 2020). Finally, inputs can also be used to induce reversible or irreversible cell differentiation (Aditya et al., 2021; Salzano, Fiore and Bernardo, 2021), which may be particularly relevant for bioproduction communities where subpopulations are focused on replication or production. Recent developments in CRISPR have enabled organism and locus-specific genome editing without the need to isolate and culture the species (Rubin et al., 2022), potentially allowing these inducible responses to be engineered into key species within a complex natural community.

While off-target effects of environmental parameters are undesirable when using inducible promoters, they can act as inputs in their own right: different species have a range of optimum pH, temperatures, osmolarities, and oxygen concentrations, and these parameters can be controlled to directly adjust selective pressures for particular species e.g., increasing pH to select for an acidophile, adding salt to select for a halophile, or displacing oxygen to select for an anaerobe. Environmental factors can also influence interspecies interactions; for example, pH may affect the degradation rate of quorum sensing molecules (You et al., 2004). In this context where response to the input is not engineered, prior knowledge of species characteristics or testing is needed to determine how the composition responds to input. Other examples of direct input that don’t induce an engineered response include the introduction of antibiotics or bacteriocins that can affect a broad range of species, or more targeted bacteriophages (Lu and Collins, 2007) that reduce the relative abundance of a subset of the community. Varying ability to utilise nutrients can also be exploited as an input: Treloar et al. (2020) proposed using different carbon sources and Martinez et al. (2022) showed that (open loop) dynamic feeding profiles with different substrates stabilised a co-culture of yeast and *E. coli*, while Fiore et al. (2022) demonstrated *in silico* simulated closed-loop control of a co-culture by modulating growth medium dilution rate. Kusuda, Shimizu and Toya (2021) as well as Bertaux et al. (2022) both successfully used cybergenetic control to tune the population ratio of essential amino acid auxotrophs by adjusting the concentration of amino acids in the media. Other chemical additives that differentially affect species such as minerals, secondary metabolites [e.g., root exudates (Zhalnina et al., 2018)], or signalling molecules (Stephens et al., 2019; Miano, Liao and Hasty, 2020) can also be used. Finally, spatial partitioning can have a large effect on cells, both influencing their local environment (factors such as pH and oxygen concentration) as well as modulating the strength of interactions between species (Timmermans et al., 2018; Dal Co et al., 2020). Partitioning has been shown to support populations with primarily negative inter-species interactions (Wu et al., 2022) while aggregate formation can promote species with mutually positive interactions (Konstantinidis et al., 2021). It can be modulated on solid media by varying the distance and arrangement of colonies (Jiang et al., 2022), or in liquid media by controlling properties such as shaking/mixing speed (shaker in Figure 2), flow rate, or available surface area (Timmermans et al., 2018).

Instead of inducing an engineered response or selecting for species by changing environmental conditions, a species’ relative abundance can also be increased by direct addition into the community. For bioprocesses, this method of input can be completely automated: Aditya et al. (2021) (discussed further below) were able to adjust the composition of a two species yeast co-culture by pumping one strain from a “reservoir” into the main culture, increasing its relative abundance. The number of reservoirs could be increased for complex communities with more species, and while maintaining several monocultures to control one community may negate economic benefits in bioproduction, the relative simplicity in implementing this input (i.e., no genetic engineering is required) means that it could be useful for probing population dynamics and gaining fundamental insights for a community. For natural communities, faecal microbiota transplants to treat *C. difficile* infections are an example of how direct addition is used to actuate community composition—the microbiota from the faeces of a healthy donor colonises the recipient gut to provide resistance against recurring infection (Sorbara and Pamer, 2022). As another example, rationally designed consortia with defined functions (e.g., production of short chain fatty acids) and traits that support colonisation have been introduced to murine gut microbiota to treat immune-mediated colitis (van der Lelie et al., 2021). While the amount of inputs increase with complexity of the community, communities can be controlled without needing to actuate every constituent species-actuating a subset of “driver species” determined from interspecies interactions can also be enough to drive the entire community to the desired state (Angulo, Moog and Liu, 2019).

Overall, the choice of input method is a trade-off between many factors such as community size and complexity (and thus the number of orthogonal inputs required), feasibility of engineering the constituent species, ease of implementation, and the level of desired or necessary control (for example, an engineered chemical-inducible response may allow for fine-tuned control of a single species within a community, while altering pH will affect a broad range of species unequally). The environment also limits possible methods of input: for example, while nutrient availability in the gut microbiome could be modulated through diet or prebiotics, spatial partitioning cannot be easily changed. Finally, a system may use several input methods in conjunction to affect community composition: for example, Connors et al. (2022) adjusted 4 parameters (pH and concentration of sugars, amino acids, and yeast extract) in an open-loop system to regulate the makeup of a 10 species community.

## Control output: Measuring community composition

Having actuated the community, the next challenge lies in measuring its response. This measured “output” is needed for comparison with the reference to calculate the error and compute the next control action that brings the error toward zero and achieve the desired community composition. A common method of measuring output in cybergenetic systems is fluorescence: both in systems controlling the expression of a fluorescent protein (Milias-Argeitis et al., 2011; Uhlendorf et al., 2012; Melendez et al., 2014, 2014; Menolascina et al., 2014; Fiore et al., 2016; Lugagne et al., 2017; Rullan et al., 2018; Shannon et al., 2020), and systems where the fluorescent protein acts as a proxy e.g., if it is linked to expression of a different gene (Toettcher et al., 2011; Perrino et al., 2019). By expressing different reporters in each species, fluorescence can also act as a proxy for community composition in cybergenetic control. The intensity of each fluorophore across the population can be measured concurrently as an approximation of the relative abundance of each species (Shou, Ram and Vilar, 2007; Kusuda, Shimizu and Toya, 2021) or by measuring fluorescence of many individual cells e.g., through flow cytometry (Aditya et al., 2021; Konstantinidis et al., 2021) or microfluidics systems with microscopes (Chait et al., 2017) (outputs in Figure 2). Fluorescence has additional benefits: it provides spatial information in heterogenous environments (Chen et al., 2015; Kozlowski et al., 2021; Krishna Kumar et al., 2021) and computational tools with integrated image analysis and control algorithms have already been developed (Pedone et al., 2021). However, it also has drawbacks as a method of measuring community composition: fluorescent intensity is an imperfect proxy for population data as there can be variations in expression levels and delays [due to folding kinetics (Pédelacq et al., 2006)] between reporters. The number of species that can be tracked is also limited by the number of fluorescent reporters with orthogonal excitation and emission spectra. While flow cytometry increases this limit by allowing species to carry more than one reporter [i.e., giving each species a unique combination (Bertaux et al., 2022)], expressing several reporters comes at the cost of increased metabolic burden.

Beyond reliance on engineered fluorescence, flow cytometry can also differentiate cells based on natural scattering properties and autofluorescence (Boddy et al., 2000; Giana et al., 2003; Bhatta, Goldys and Learmonth, 2006) or fluorescence from universal dyes, which can potentially even distinguish different strains of the same species. For example, Boon et al. (2018) was able to differentiate between a synthetic community of 27 strains of *Lactobacillus* (representing 8 distinct species) through staining with SYBR Green I and propidium iodide. This ability to measure composition without engineering the constituent species may be particularly important in environments where genetically modified organisms are impractical or undesirable e.g., when looking to control natural communities. Flow cytometry has already been used to monitor subcommunities within large, complex communities in wastewater (Koch et al., 2013), freshwater (Props et al., 2016), or maize silage (Lambrecht et al., 2018). Other advantages include its ability to be automated (Milias-Argeitis et al., 2016) and the existence of tools such as flowEMMi (Ludwig et al., 2019) and the CellCognize pipeline (Özel Duygan et al., 2020) that have been developed to translate cytometric data into microbial population data. However, while flow cytometry excels at characterising “known” populations (e.g., in a synthetic community) (Özel Duygan et al., 2020; Aditya et al., 2021; Bertaux et al., 2022; Gutiérrez, Kumar and Khammash, 2022; Martinez et al., 2022), increased complexity can result in an inability to differentiate, or misclassification of species (Özel Duygan et al., 2020).

For more complex communities, accurate, high-resolution measurement of composition can instead be achieved through sequencing (sequencing read in Figure 2) of the whole genome or amplicon sequencing of marker genes such as 16s (Johnson et al., 2019), where a universal primer is used to amplify the 16s gene from every species in the community. Alternatively, artificial markers such as plasmid barcodes can be engineered into strains: Wu et al. (2022) used sequencing to quantify population dynamics of a 47-strain community of plasmid-barcoded *E. coli*. While marker gene sequencing may not provide functional information for more in-depth community study, its reduced cost and high resolution make it well suited for real-time monitoring of relative abundance (Benítez-Páez, Portune and Sanz, 2016), though on its own it cannot provide absolute abundance data (Jian et al., 2020). Developments in third generation sequencing technologies such as the nanopore technologies continue to decrease time and cost per base pair, leading to the rise of *in situ* metagenomic sequencing as reviewed by (Latorre-Pérez et al., 2020) who also highlighted their potential use in real-time monitoring of industrial bioprocesses to provide feedback and enable corrective actions. However, the relative cost of sequencing (particularly for longer reads covering the entire 16s gene instead of smaller variable regions), lower throughput (requiring DNA extraction and amplification), and slow timeframe remain drawbacks compared to methods such as fluorescence and flow cytometry.

Less common methods to measure composition include: dilution plating on different selective medias and counting CFUs (colony forming units) (Wu et al., 2022), which is laborious and slow; qPCR (quantitative Polymerase Chain Reaction) of unique DNA sequences (Mee et al., 2014; Jian et al., 2020; Kleyer, Tecon and Or, 2021); or RNA sensors on paper-based cell-free systems (Takahashi et al., 2018). qPCR and RNA sensors can be quantitative and cost effective, but typically require primer/RNA sensor design for each species and involve laborious sample extraction and amplification. For communities in locations where frequent sampling is difficult or inconvenient such as the human gut, species abundance can be monitored non-invasively using bioluminescent imaging (Foucault et al., 2010) or acoustic reporter genes, which encode gas vesicles that can be detected through ultrasound (Bourdeau et al., 2018; Sawyer et al., 2021). While promising, both are limited by the number of orthogonal reporters, though continued development and genome mining efforts may lead to advances like those of fluorescent proteins (Hurt et al., 2021).

Overall, when choosing a measurement approach, characteristics such as measurement frequency and delay, cost, automatability, ease of sampling, community size, and requirement to engineer each species in the community should be considered, and multiple methods can be used in tandem to complement one another (Table 1). For example, a synthetic four species community of easy-to-engineer species in a well-characterised and tightly controlled bioproduction environment (Jones et al., 2017) can rely on fluorescence for constant, immediate, and automated population data collection at very little cost. In comparison, a complex community in a natural environment cannot expect to have the same sampling frequency, but as suggested by (Props et al., 2016), can still achieve accurate high frequency measurements through flow cytometry with results supported by supervised 16s sequencing at less frequent intervals (i.e., supervised sampling at points of interest, such as when flow cytometry indicates large changes in relative abundance). Though the delay between measurement and useful data is significantly longer, it still “closes the loop”, providing feedback that can shape the input.

**TABLE 1:** Methods of measuring community composition (output) and their characteristics (i.e. factors that need to be considered when selecting a method).

Method	Requires species to be engineered	Maximum community size	Timeframe	Equipment required	Commonly automated	Quantitative	Other considerations
Community averaged fluorescence (e.g., plate reader)	Yes	Limited by number of orthogonal fluorescent proteins	Seconds	Fluorescent spectrometer or plate reader	Yes	Yes	• Imperfect proxy for population (e.g., different folding kinetics) • Does not account for population heterogeneity
Single cell fluorescence through microscopy (e.g., with microfluidics/mother machine)	Yes	Limited by number of orthogonal fluorescent proteins	Minutes	Microscope	Yes	Yes	•Provides spatial data
Single cell fluorescence through flow cytometry	Yes	Limited by number of orthogonal fluorescent proteins	Minutes	Flow cytometer	Yes	Yes	• Can measure multiplexed fluorescent reporters (>1 fluorophore per cell), allowing more possible combinations
Flow cytometry with autofluorescence	No	Dependent on community	Minutes	Flow cytometer	Yes	Yes	• May not provide strain/species level resolution
Flow cytometry with dyes and natural scattering	No	Dependent on community	Minutes-hours	Flow cytometer	No	Yes	• May not provide strain/species level resolution
Marker gene (e.g., 16s) sequencing	No	Extremely large, complex communities	Hours-days	Sequencer or sequencing service	No	No (relative abundance only)	• Little prior knowledge of species required; • Biases from PCR amplification step • Labour intensive • Limited strain level resolution
Barcode sequencing	Yes	Can be designed for community of any size	Hours-days	Sequencer or sequencing service	No	Yes (requires calibration)	• Can account for biases from PCR amplification• Requires calibration to be quantitative • Labour intensive
Selective plating and counting cfus (colony forming units)	Yes	Limited by selection pressures	Days	Selective plates	No	Yes	• Extremely labour intensive • Species must be culturable
qPCR (quantitative polymerase chain reaction) of unique DNA sequences	Yes	Can be designed for community of any size	Hours	Real-time PCR thermocycler	No	Yes	• PCR is labour intensive
Cell free RNA sensors	No	Can be designed for community of any size	Hours	Paper-based RNA sensor	No	Semi quantitative	• Can target 16s genes • Design and optimisation required (e.g., NASBA primers)• Each sensor must be tested against all others for orthogonality
ARGs (acoustic reporter genes)	Yes	2 orthogonal outputs	Hours	Ultrasound scanner	No	Yes	• Allows non-invasive imaging in hard-to-reach communities • Can image single cells• Provides spatial data

## Control algorithms: Designing the control approach

Once the output is measured, a control algorithm (Table 2) determines an appropriate input level to drive the community to the desired composition. Given the same system, different algorithms can lead to drastically different outcomes (Fiore et al., 2016; Milias-Argeitis et al., 2016; Lugagne et al., 2017), and hence algorithm selection depends on the application and control goals in question. An easy-to-design and deploy algorithm is bang-bang control (Lugagne et al., 2017; Carrasco-López et al., 2020), which simply selects a binary control action (the input to the community, e.g., light/no light) based on the sign of the error (i.e., species’ relative abundance is above/below a desired threshold). For certain input methods, binary control actions may be preferred over continuous techniques for ease of implementation (e.g., providing or withholding a chemical inducer is simpler than providing a range of concentrations), but control actions from other algorithms with superior characteristics can be converted from continuous to discrete bang-bang through pulse width modulation in some cases (Menolascina et al., 2014).

**TABLE 2:** Control algorithms that can be used for cybergenetic control and their respective advantages/disadvantages.

Control Algorithms	Advantages	Disadvantages
Bang-Bang	• Model not required	• Poor at handling delays in system
	• Easy to implement computationally	• SISO (single input single output) system that only controls one species
	• Qualitative inputs may be easier to implement (e.g., inducer/no inducer vs. a range of inducer concentrations)	
PI (proportional-integral)	• Model not required	• SISO (single input single output) system that only controls one species
	• Easy to implement computationally	• Cannot vary reference over time
		• Susceptible to windups and oscillatory dynamics
MPC (model predictive control)	• Can vary reference over time	• Model required
	• Can be applied to MIMO (multiple input multiple output) systems that control >1 species	• Optimisation required to fit experimental data to model
		• Computationally expensive
ZAD (zero average dynamics)	• Can vary reference over time	• Model required
		• Optimisation required to fit experimental data to model
Reinforcement learning	• Model not required	• Requires more training data than MPC
		• “Black box” system and difficulty in extrapolating past training data set conditions

One of these alternative algorithms is PID (Proportional-Integral-Derivative) control, a classic control algorithm commonly implemented across engineering and in particular cybergenetics. The control action is the sum of a term proportional to the current error (the “P” term), a term that is the integral of the past error (“I” term), and a term that is the derivative of the error (the “D” term), but depending on the system suitable control may be achieved with just P-control (Kusuda, Shimizu and Toya, 2021), just I-control (Rullan et al., 2018), PI-control (Toettcher et al., 2011; Menolascina et al., 2014; Fiore et al., 2016; Milias-Argeitis et al., 2016; Lugagne et al., 2017), or PID-control (Gutiérrez, Kumar and Khammash, 2022). PID-type controllers are particularly desirable as they are computationally simple to implement and do not require a model or deep understanding of the biological system for tuning (Milias-Argeitis et al., 2016), though model-guided optimisation and tuning of controller parameters such as gains allow for improved control and optimisation of different targets for different applications (Gutiérrez, Kumar and Khammash, 2022). Given a constant reference community composition, PID control is capable of driving the error to zero and performing disturbance rejection for many common classes of disturbance, maintaining the community at a desired reference, as demonstrated in a two-species co-culture of auxotrophic *E. coli* strains controlled by amino acid concentration (Kusuda, Shimizu and Toya, 2021). However, there are drawbacks to PID control: it functions best at set-point control but is less effective if the reference is dynamic (Menolascina et al., 2014), is susceptible to windups and oscillatory dynamics (Fiore et al., 2016), and is typically best suited for SISO (Single Input Single Output) systems while complex communities may need multiple input methods to actuate all the species (or have each species controlled by an individual PI controller).

MPC (Model Predictive Control) is another common alternative for cybergenetics (Milias-Argeitis et al., 2011, 2016; Uhlendorf et al., 2012; Fiore et al., 2016; Chait et al., 2017; Perrino et al., 2019; Aditya et al., 2021; Bertaux et al., 2022) that overcomes some of the issues of PID control. It predicts system behaviour over a finite time interval using a model of the system, alongside the current output and reference to decide the input, and then repeats this process when data is subsequently collected (Morari and H. Lee, 1999). MPC has been shown to effectively follow a reference that ramps down linearly, decreases in step values, or follows a sine wave in a biological system (Fiore et al., 2016), can be applied to MIMO (Multiple Input Multiple Output) systems (Tanaskovic et al., 2013), and the iterative nature means that modelling inaccuracies are not propagated in time (Milias-Argeitis et al., 2016). However, MPC is more computationally expensive and requires accurate mathematical models, necessitating prior knowledge or preliminary system identification experiments to determine model parameters (Milias-Argeitis et al., 2011) and additional experiments whenever the plant is altered (e.g., a new species is added). As the complexity of the community increases, it also becomes increasingly difficult to accurately model the first and higher order interactions between all species.

Other control strategies include ZAD (Zero Average Dynamics) which is also model-based and demonstrated similar results to MPC (Menolascina et al., 2014), and deep reinforcement learning combined with bang-bang inputs (Treloar et al., 2020). The latter was shown to be superior to PID for *in silico* simulated population control of a co-culture of auxotrophic bacteria, particularly when composition sampling was less frequent, making it more suitable when using methods of measuring output community composition which are expensive and slow. Reinforcement learning approaches are also model-free, though they still require experiments to gather training data. Fiore et al. (2022) also showed *in silico* co-culture population control using a gain-scheduling state feedback controller and a sliding controller, both of which altered dilution rate of a chemostat as an input. Overall, when choosing a control strategy (Table 2), factors to consider include: the overall length of time needed to design and optimise the approach, which is heavily influenced by the need for a system model, the control goal (i.e., whether set point control with a fixed reference is sufficient), the capabilities of the input method, the frequency of measured output and the time delay between measurement/actuation, and the computational cost.

## Cybergenetic community control: Recent examples

The application of cybergenetics to control of microbial community composition has not been reported as frequently as cybergenetic control of gene expression. As of publication of this review, the authors are aware of four completely closed-loop experimental systems, with several others that have input and output methods but do not use the feedback to close the loop. The complete systems are described briefly below.

In 2021, Kusuda, Shimizu and Toya displayed cybergenetic control of continuous *E. coli* co-culture in a custom Arduino-based 1-L reactor. The strains were methionine or arginine auxotrophs, and the culture was supplied with either plain media or media supplemented with either amino acid as the input method. The strains carried either Green or Red Fluorescent Protein (GFP/RFP), allowing their relative abundance to be monitored *in situ* through community-averaged fluorescence using LEDs and light sensors on the device. With just Proportional control (P-control), they were able to maintain a defined population ratio for >25 h. As the authors point out, their setup could easily be adapted for strains which utilise different carbon sources or expanded to control more than two strains. However, they also concur that they only maintain a static setpoint instead of a dynamic reference and that measurement of composition could be more robust, as their results assumed that difference in culture parameters did not affect fluorescence characteristics (for example, cells in stationary phase that are starved of their respective amino acids are unlike to fluoresce at the same intensity as cells in exponential phase with an excess of amino acids).

In a 2021 preprint followed by a peer reviewed publication in 2022, Bertaux et al. reported the ReacSight strategy, which amongst other capabilities was able to dynamically control composition of a two species yeast co-culture in a custom made 30 ml reactor or a commercially available 30 ml Chi.bio reactor (Steel et al., 2020). The co-culture consisted of a histidine auxotroph and a histidine prototroph (i.e., able to produce histidine) with a slow-growth phenotype. Auxotroph growth was limited by histidine availability, and the system grew the co-culture using media with a fixed concentration of histidine. As histidine availability was determined by the OD setpoint (since higher ODs led to increased nutrient consumption and thus decreased histidine availability), the input to the system was adjustments to the OD setpoint, which led to changes in growth rate of the histidine auxotroph relative to the prototroph. The output was single-cell fluorescence of mCerulean and/or mScarlet measured through automated flow cytometry, achieved with a pipetting robot and ReacSight (Bertaux et al., 2022). With Model Predictive Control (MPC) they were able to control community composition but encountered issues with steady state error and then oscillations, which were attributed to time delays that were not accounted for in the model.

Later in 2021, Aditya et al. demonstrated cybergenetic control over differentiated and non-differentiated yeast cells in continuous culture inside the ReacSight reactors (Bertaux et al., 2022). They initially combined two input methods, using optogenetics to induce genetic recombination that leads to differentiation (and thus increase relative abundance of differentiated cells) or pumping of non-differentiated cells from a “reservoir” reactor into the main reactor (to increase relative abundance of non-differentiated cells), and then inserted an optogenetic growth arrest system into differentiated cells that could be induced to allow non-differentiated cells to outcompete, eliminating the need for the reservoir. Similar to before, the output was single-cell fluorescence of mCerulean or mNeonGreen using automated flow cytometry and ReacSight (Bertaux et al., 2022). Using MPC they demonstrated control with a dynamic (reservoir reactor as an input) and static (growth arrest as an input) reference, and then further expanded the system by cloning two recombination cassettes into the non-differentiated strain, creating sub-populations upon light induction. However, while composition of the sub-populations was affected by the duration of light pulses, they did not demonstrate control over this consortia.

Finally, in 2022, Gutiérrez, Kumar and Khammash demonstrated cybergenetic control over an *E. coli* co-culture in a commercially available 40 ml eVOLVER reactor (Wong et al., 2018) which contained constant, sub-lethal levels of an antibiotic for up to 40 h. One strain constitutively expressed the antibiotic resistance gene (and thus had a fixed growth rate) while a photophilic (i.e., thrives in light) strain carried a light-inducible resistance gene, allowing dose response of its growth rate to blue light, which acted as the input to this system. Similarly, the output was measured through automated flow cytometry, with either presence or absence of the mVenus fluorescent reporter. This system employed PID control and was able to accurately drive the composition to static and dynamic references, steering the co-culture to arbitrary population ratios over a long period of time. While PID can be implemented without a model of the system, this work also demonstrated that more robust control can be obtained by optimising PID parameters through the use of models.

## Conclusion

Cybergenetic control of protein expression has provided researchers with many insights into gene networks and regulatory dynamics (Perrino et al., 2019). Similarly, there is much to be gained if microbial communities were controlled and probed in the same manner, taking advantage of computers to perform tasks that biology cannot efficiently perform. A wide variety of methods for actuating species in a community through direct and indirect means as well as methods of measuring community composition already exist. However, since communities used in different applications differ significantly in species and context (e.g., number of species, environment), there is no optimal one-size-fits-all approach to controlling community composition. Therefore future work will likely include combining existing methods of control input and output in novel approaches in addition to refining current strategies. Continued improvement of input (e.g., development of more orthogonal inducers) and output (e.g., decreasing sequencing costs or improving flow cytometry accuracy) methods are also enabling technologies that will advance the field. Outside of the cybergenetic strategy, there is also the need to demonstrate robust cybergenetic control of more than two species, taking advantage of the fact that *in silico* control circuity can compute the complex logic needed to control larger communities. As the technology matures, there is also significant potential in moving past the proof-of-concept stage and applying demonstrated or novel setups to biotechnological or ecological applications.

Real-time monitoring and cybergenetic control of microbial communities will enable the adoption of defined communities in place of monocultures in many applications, establishing a new paradigm for bioproduction and bioremediation through synthetic communities. Its application towards complex natural communities will also enable a better understanding of their population dynamics and potentially provide a measure of control over these important systems that are central to fields ranging from biomedicine to agriculture.
